# Cancer stem-like cells can be induced through dedifferentiation under hypoxic conditions in glioma, hepatoma and lung cancer

**DOI:** 10.1038/cddiscovery.2016.105

**Published:** 2017-01-23

**Authors:** Pan Wang, Wen-wu Wan, Shuang-Long Xiong, Hua Feng, Nan Wu

**Affiliations:** 1 Department of Neurosurgery, Southwest Hospital, Third Military Medical University, Chongqing 400038, China; 2 Department of Oncology, Cancer Hospital, Chongqing 400030, China

## Abstract

Traditional studies have shown that transcription factors, including SOX-2, OCT-4, KLF-4, Nanog and Lin-28A, contribute to the dedifferentiation and reprogramming process in normal tissues. Hypoxia is a physiological phenomenon that exists in tumors and promotes the expression of SOX-2, OCT-4, KLF-4, Nanog and Lin-28A. Therefore, an interesting question is whether hypoxia as a stimulating factor promotes the process of dedifferentiation and induces the formation of cancer stem-like cells. Studies have shown that OCT-4 and Nanog overexpression induced the formation of cancer stem cell-like cells through dedifferentiation and enhanced malignancy in lung adenocarcinoma, and reprogramming SOX-2 in pancreatic cancer cells also promoted the dedifferentiation process. Therefore, we investigated this phenomenon in glioma, lung cancer and hepatoma cells and found that the transcription factors mentioned above were highly expressed under hypoxic conditions and induced the formation of spheres, which exhibited asymmetric division and cell cycle arrest. The dedifferentiation process induced by hypoxia highlights a new pattern of cancer development and recurrence, demonstrating that all kinds of cancer cells and the hypoxic microenvironment should be taken into consideration when developing tumor therapies.

## Introduction

Dedifferentiation, as a universal biological phenomenon, involves the regression from a specialized differentiated tissue to a stem cell-like state with retained self-renewal properties. Stem cells, particularly embryonic stem cells, have had a vital role in degenerative diseases and regenerative medicine but remain an issue of ethical morality, and their use in studies is thus controversial. To avoid this, researchers have successfully induced the formation of pluripotent stem cells (iPSCs) from adult fibroblasts or other somatic cells using defined transcription factors, including SOX-2, OCT-4, KLF-4, Nanog, LIN-28A and C-MYC.^1–4^ Hence, these transcription factors contribute significantly to the dedifferentiation process in normal tissues. However, an interesting question is what the influences of these factors on cancer stem cells (CSCs) are. CSCs, as another type of stem cells, have been shown to contribute to tumor recurrence, resistance to chemo- and radiotherapy and malignant development.^5,6^ Recently, a series of studies demonstrated that these transcription factors are highly expressed in glioma,^7^ pancreatic cancer,^8,9^ breast cancer,^10^ lung adenocarcinoma^11^ and hepatoma.^12^ In 2010, Chiou *et al.*^11^ found that the overexpression of OCT-4 and Nanog promoted the formation of spheres with an increase of CD133 in lung adenocarcinoma. A similar process was detected in pancreatic cancer^8^ and colon cancer,^13^ both defined by SOX-2 and Lin-28B. Therefore, these transcription factors are also vital and necessary for the dedifferentiation process of tumors, thus inducing the formation of cancer stem-like cells.

In induced pluripotent stem cells (iPSCs), findings have revealed that the reprogramming efficiency significantly increased after hypoxia exposure,^14^ indicating that hypoxia has an important role in the dedifferentiation process. A tumor is an abnormal tissue in the body that grows without control. In addition, the proliferation rate of tumor cells is much faster than the formation of vessels, thus promoting the formation of a hypoxic microenvironment around tumor cells, including cancer stem cells and non-cancer stem cells (non-CSCs).^15^ Traditional studies have shown that hypoxia has a critical role in the stemness maintenance of CSCs. However, many researchers have ignored the influence of hypoxia on non-CSCs. Thus, to date, there have been few studies concentrated on non-CSCs influenced by hypoxia as a sole factor. In 2013, Li *et al.*^15^ found that unsorted glioma cells highly expressed SOX-2, OCT-4 and CD133 after hypoxia exposure, which is in accordance with a study carried by Blazek^16^ in 2007. However, these studies cultured unsorted tumor cells under hypoxia in a stem cell medium containing EGF and FGF2; thus, it was difficult to determine which types of cells (CSCs or non-CSCs) facilitated the expansion of CSCs. Moreover, EGF and FGF2, as growth factors, can promote the dedifferentiation process;^17,18^ thus, it is still not possible to determine the effects of hypoxia on non-CSCs. To rule out these factors, we sorted tumor cells at least three times and performed our experiments in DMEM/F12 medium with 1% FBS, and we have taken glioma, hepatoma and lung cancer into consideration and detected dedifferentiation under hypoxic conditions.

## Results

### Hypoxia increased the expression of transcription factors and stem cell markers

Studies have shown that transcription factors such as SOX-2, OCT-4, KLF-4, Nanog and Lin-28A contribute to tumor cell dedifferentiation.^7,8,10,11,13^ Therefore, we first detected whether hypoxia promoted an increase in SOX-2, OCT-4, KLF-4, Nanog and Lin-28A expression at the mRNA and protein levels.

**Figure 1:**
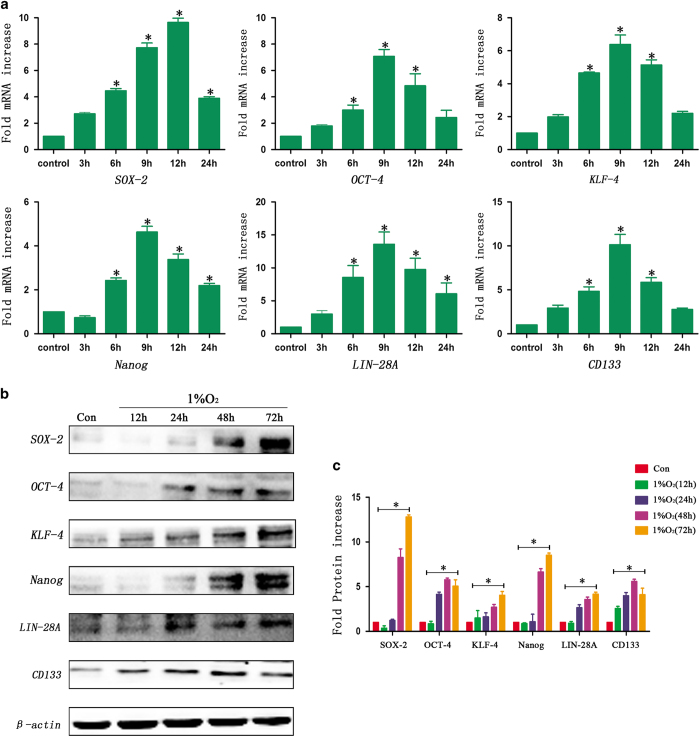
Hypoxia promoted an increase in the expression of SOX-2, OCT-4, KLF-4, Nanog, Lin-28A and CD133. (**a**) The RT-qPCR analysis showed an upregulation in the expression of SOX-2, OCT-4, KLF-4, Nanog, Lin-28A and CD133 in a time-dependent manner in sorted A549 cells under hypoxic conditions (*P<0.05). For SOX-2, the peak expression was after 12 h of hypoxia, and the expression then decreased slightly but remained statistically higher than that of the control cells. The expression of OCT-4, KLF-4, Nanog Lin-28A and CD133 was highest after 9 h of hypoxia. (**b**, **c**) Western blot analysis showed that the transcription factors and stem cell markers were not expressed under normoxia. However, there was an increase in the expression of SOX-2, OCT-4, KLF-4, Nanog, Lin-28A and CD133 in a time-dependent manner after hypoxia treatment in sorted A549 cells (*P<0.05).

First, RT-qPCR was used to detect mRNA after hypoxia exposure. We found that these transcription factors were not expressed under normoxic conditions (21% O_2_) in sorted A549 cells but that the expression of these factors increased significantly in a time-dependent manner following hypoxia treatment for 3, 6, 9, 12 and 24 h. In addition, the results showed that the peak expression of SOX-2 was after 12 h of the hypoxia treatment, but the highest expression of OCT-4, KLF-4, Nanog and Lin-28A was observed after 9 h of hypoxia (Figure 1a). The expression of CD133, a stem cell marker in A549 cells, also increased obviously under hypoxic conditions (Figure 1a). Similar results were observed in sorted GL261 (Supplementary Figure S1A) and HepG2 cells (Supplementary Figure S1B).

Second, the results of the western blot analysis showed that there was no expression of SOX-2, OCT-4, KLF-4, Nanog and Lin-28A in sorted A549 cells under normoxic conditions. However, the expression increased remarkably in a time-dependent manner following hypoxia treatment for 12, 24, 48 and 72 h (Figures 1b and c). In addition, the CD133 expression in sorted A549 cells also increased after hypoxia exposure, and the peak expression was after 48 h of hypoxia. Similar results were identified in sorted GL261 (Supplementary Figure S2A) and HepG2 cells (Supplementary Figure S2B).

**Figure 2:**
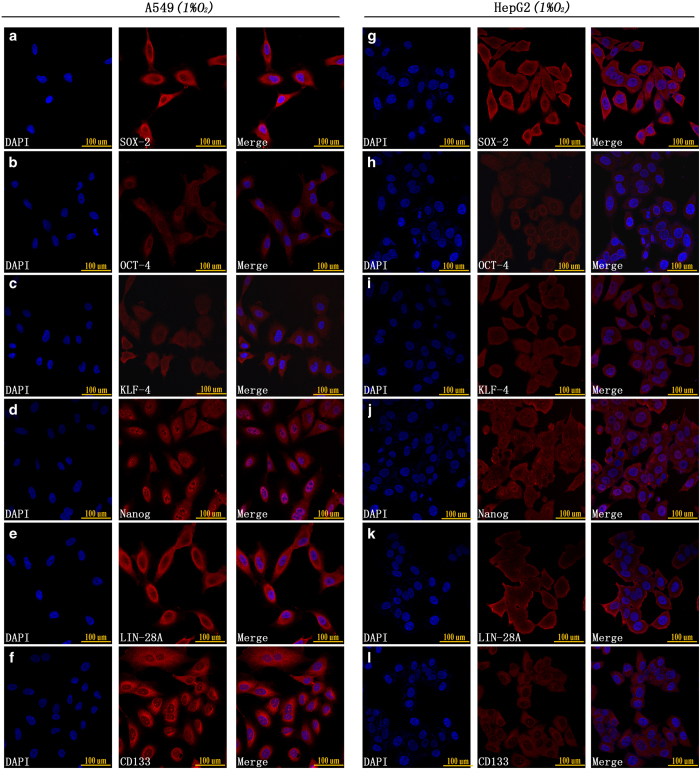
(**a**–**f**) Immunofluorescence staining showed that A549 CD133-negative cells highly expressed SOX-2, OCT-4, KLF-4, Nanog, Lin-28A and CD133 after 48 h of 1% O_2_ exposure. (**g**–**l**) Immunofluorescence staining showed that HepG2 CD133-negative cells highly expressed SOX-2, OCT-4, KLF-4, Nanog, Lin-28A and CD133 after 48 h of 1% O_2_ exposure.

In addition to RT-qPCR and western blot analyses, we used immunofluorescence to detect the expression of stem cell markers after 48 h of hypoxia exposure. The results showed that both A549 and HepG2 cells highly expressed SOX-2, OCT-4, KLF-4, Nanog, Lin-28A and CD133 under hypoxic conditions (Figure 2).

### Hypoxia increased the expression of putative CSC markers

**Figure 3:**
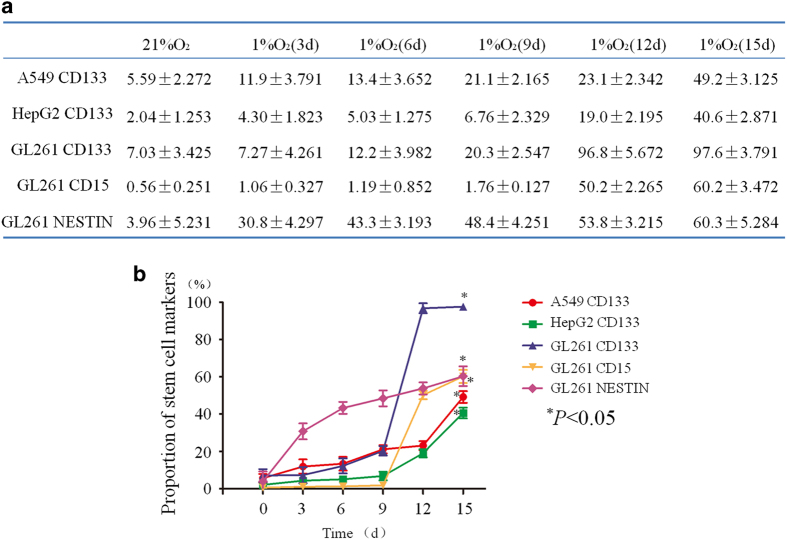
Hypoxia increased the expression of putative CSC markers. (**a**) In sorted A549 cells, the proportion of CD133-positive cells was only 5.59%±2.272 in the control cells, and this proportion increased in a time-dependent manner under hypoxic conditions. After 15 days of hypoxia, the proportion of CD133-positive cells reached 49.2%±3.125. The CD133 expression in sorted HepG2 cells also increased from 2.04%±1.253 to 40.6%±2.871 after 15 days of hypoxia. For GL261 cells, the proportion of CD133-positive cells was 7.03%±3.425 in the normoxia control cells, and this rate increased remarkably from 9 days to 15 days of hypoxia exposure from 20.3%±2.547 to 97.6%±3.791. In addition, the results also showed that the expression of two other stem cell markers, CD15 and NESTIN, was upregulated, increasing from 0.56%±0.251 to 60.2%±3.472 and 3.96%±5.231 to 60.3%±5.284, respectively, after 15 days of hypoxia. (**b**) The proportion of stem cell markers in sorted cells increased significantly under hypoxic conditions (*P<0.05).

Next, we detected the changes in the expression of stem cell markers in sorted non-CSCs (GL261 CD133^−^/CD15^−^/NESTIN^−^ cells, A549 CD133^−^ cells and HepG2 CD133^−^ cells) at 3, 6, 9, 12 and 15 days of hypoxia exposure by flow cytometry. In sorted A549 cells, the proportion of CD133-positive cells was only 5.59%±2.272 in the control cells, and this proportion began to increase in a time-dependent manner after hypoxia exposure. After 15 days of hypoxia exposure, the rate of CD133-positive cells reached 49.2%±3.125 (Figure 3a). The same trend was observed in sorted HepG2 cells after hypoxia treatment (Figure 3a). For sorted GL261 cells, the proportion of CD133-positive cells was 7.03%±3.425 in the normoxia control group, and this rate increased remarkably from 20.3%±2.547 to 97.6%±3.791 at days 9 and 15 of hypoxia, respectively (Figure 3a), demonstrating that almost all the sorted GL261 cells after 15 days of hypoxia were CD133 positive. In addition, the results also showed that the expression of two other stem cell markers, CD15 and NESTIN, was upregulated, increasing from 0.56%±0.251 to 60.2%±3.472 and 3.96%±5.231 to 60.3%±5.284, respectively (Figure 3a). These data show that the proportion of stem cell markers in non-CSCs increased under hypoxic conditions (Figure 3b).

### Sphere formation from a single sorted cancer cell was induced by hypoxia

**Figure 4:**
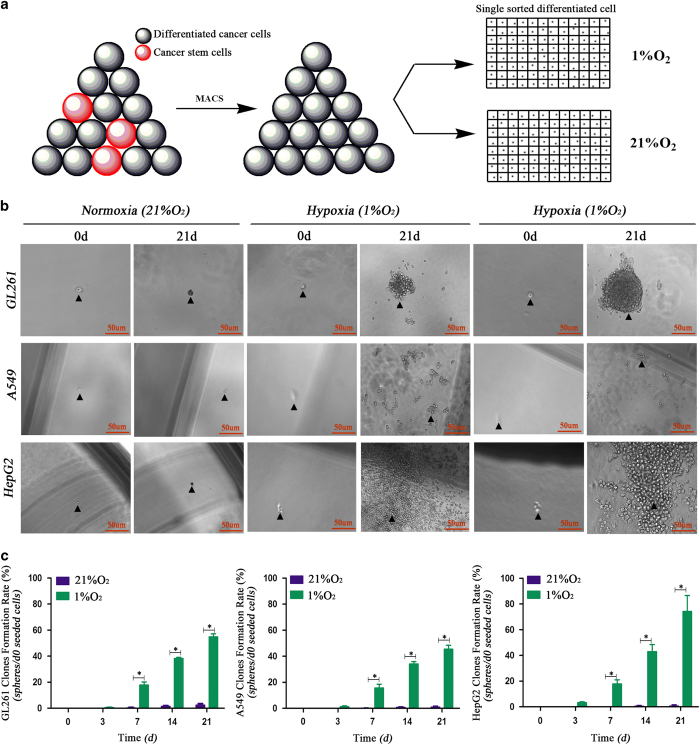
The spheres formed by single sorted cancer cells under hypoxia. (**a**) Single cancer cell seeding model; non-cancer stem cells were sorted using magnetic cell sorting, counted and diluted to 1500 cells/1 ml DMEM/F12+10% FBS, and then 1 *μ*l of the suspension was seeded into each well of 96-well plates containing 170 *μ*l of DMEM/F12 without serum. (**b**, **c**) Single sorted (CD133^−^ cells for A549 and HepG2, CD133^−^CD15^−^NESTIN^−^ cells for GL261) cancer cells formed a sphere after 21 days of hypoxia exposure; however, the cells under normoxia were dead, and there was no sphere formation (*P<0.05).

As floating sphere-like proliferation is a basic feature of CSCs,^19^ it was necessary to investigate whether hypoxia induces sphere formation in sorted cells. The results showed that sorted A549, HepG2 and GL261 non-CSCs formed spheres under hypoxic conditions, and the rates at 21 days were over 50% (sphere rate=d21 sphere number/d0 seeded cells). However, under normoxic conditions (21% O_2_), tumor cells only formed loose aggregates with a rate less than 5%, and most of the cells were dead after the normoxia treatment (Figures 4b and c).

### Stem cell markers and asymmetric division detection for newly formed spheres

**Figure 5:**
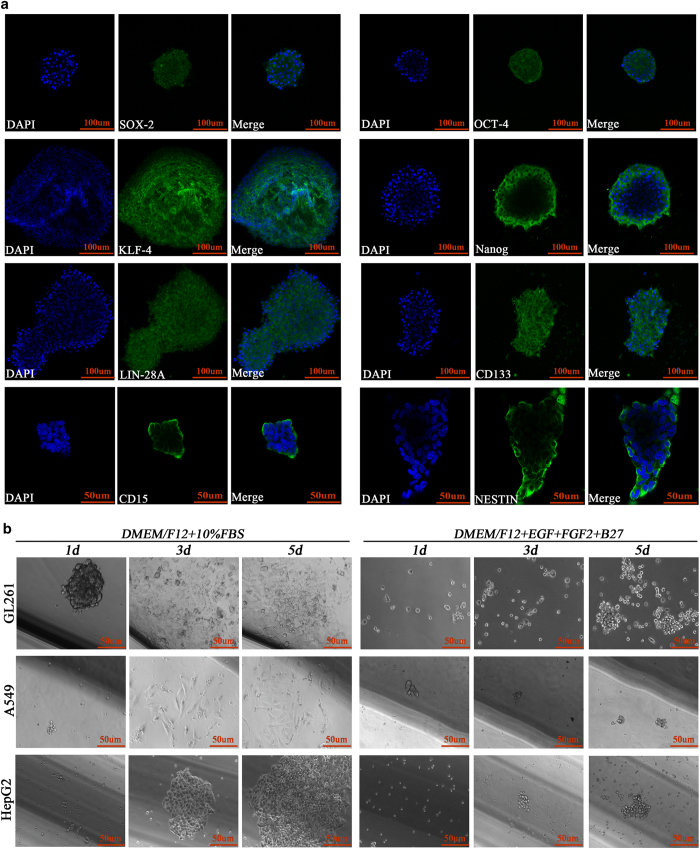
Newly formed spheres highly expressed transcription factors and showed asymmetric division. (**a**) Immunofluorescence staining showed that the newly formed GL261 spheres highly expressed SOX-2, OCT-4, KLF-4, Nanog, Lin-28A, CD133, CD15 and NESTIN. (**b**) These newly formed GL261 spheres kept growing in a suspension and proliferated extensively in stem cell culture medium (DMEM/F12+EGF+FGF2+B27) but presented an adherent phenotype in differentiated medium (DMEM/F12+10% FBS).

We used immunofluorescence to detect whether the newly formed spheres expressed stem cell markers, and the results showed that all the markers, including SOX-2, OCT-4, KLF-4, Nanog, Lin-28A, CD133, CD15 and NESTIN were highly expressed in the newly formed GL261 spheres (Figure 5a).

Asymmetric division as one of the most important features of stem cells.^20^ To evaluate whether these newly formed spheres exhibited this characteristic, we transferred these spheres to 24-well plates and cultured them in either serum-free medium containing EGF and FGF2 (stem cell medium) or DMEM/F12+10% FBS (differentiated cell medium). The results showed that these newly formed spheres exhibited self-renewal and extensive proliferation as suspension cells in the stem cell medium, but adherent growth and morphology was induced with 10% FBS administration (Figure 5b).

### Effect of hypoxia on the cell cycle and apoptosis

**Figure 6:**
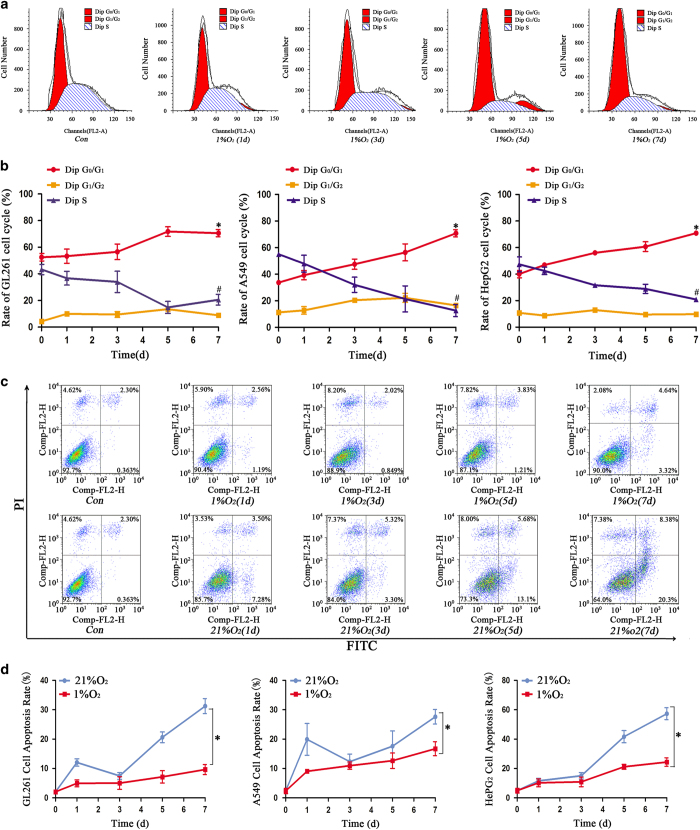
Cells under hypoxia showed cell cycle arrest and lower cell apoptosis. (**a**) A representative graph of the cell cycle arrest of sorted GL261 cells after hypoxia treatment. (**b**) Sorted GL261, A549 and HepG2 cells in hypoxia exhibited an increase in cells in the G_0_/G_1_ phase and a decrease in the proportion of cells in the G_1_/G_2_ and S phase (*P<0.05, ^#^*P*<0.05). (**c**) Flow cytometry detected the apoptosis of sorted GL261 cells under hypoxia conditions. (**d**) The cell apoptosis rate was much higher in cells treated with normoxia than those treated with hypoxia (*P<0.05).

Cell cycle arrest and apoptosis resistance are CSC characteristics.^15^ We used flow cytometry to detect the influence of hypoxia on the cell cycle and apoptosis. The results showed that more cells were arrested in the G_0_/G_1_ phase and fewer cells were in the G_2_/M+S phase after 3 days of hypoxia treatment (Figures 6a and b). In addition, we found that normoxia induced more cell apoptosis compared with hypoxia (Figures 6c and d). Besides, the newly formed spheres in hypoxia 14 days were also arrested in the G_0_/G_1_ phase (Supplementary Figure S2C). Both control cells and spheres originated under hypoxia were exposed to temozolomide (100 *μ*M) under normoxia conditions for 48 h followed by apoptosis detection through flow cytometry. Our results indicated that the cells from hypoxia-derived spheres showed reduced apoptosis compared with control normoxic cells after temozolomide treatment (Supplementary Figure S2D).

## Discussion

Dedifferentiation, as a biological phenomenon, exists in various systems, including muscle,^21^ monocytic^22^ and spermatogonial^23^ cells, which suggests that stem-like cells with both the functional and morphological properties of stem cells can be induced from general tumor cells in some cases.^24,25^ In addition to normal tissues, some tumors such as glioma, lung adenocarcinoma, pancreatic cancer and breast cancer also exhibit dedifferentiation in some cases.^26^ In 2014, Auffinger *et al.*^27^ found that therapeutic doses of temozolomide (TMZ) consistently increased the expression of pluripotency markers of glioma stem cells (GSCs), including CD133, CD15, NESTIN, SOX-2 and OCT-4, which was observed as a reversion or dedifferentiation process from non-GSCs to GSCs. To further verify this result, they implanted glioma cells after TMZ treatment into nude mice, and more efficient grafting and invasive phenotype of cells were observed. Taken together, their findings suggested that glioma cells exposed to TMZ chemotherapeutic agents were able to interconvert between non-GSCs and GSCs through dedifferentiation and differentiation. This was in accordance with a study conducted by Dahan *et al.*,^28^ which demonstrated that ionizing radiation treatment can induce the formation of GSCs through dedifferentiation, with an increase in the expression of stem cell markers such as CD133, SOX-2, NESTIN and Olig2 and a decrease in the expression of the differentiated markers GFAP and Tuj1. Moreover, the identified cancer cells after ionizing radiation treatment possessed a high ability to generate primary and secondary neurospheres with increased tumorigenicity. In addition, DNA damage inducers such as UV light and mitomycin C also increased the number of CSCs in NPC CNE-2 and neuroblastoma SKN-SH cells.^29^

Many studies have shown that transcription factors, such as SOX-2, OCT-4, KLF-4, Nanog and Lin-28A, have an important role in dedifferentiation or reprogramming processes. Herreros-Villanueva *et al.*^8^ reprogrammed SOX-2 in pancreatic cancer cells, and the results showed that reprogramming the cells promoted cell proliferation and contributed to stemness/dedifferentiation. This was in accordance with research by Chiou *et al.*,^11^ whose study demonstrated that the co-expression of OCT-4 and Nanog promoted the formation of cancer stem cell-like cells through dedifferentiation and enhanced malignancy in lung adenocarcinoma. A similar phenomenon was also detected in ovarian cancer recently, and this study showed that both primary and recurrent ovarian cancer highly expressed OCT-4A and that the knockdown of OCT-4A reduced cell proliferation, inhibited spheroid formation, suppressed stem cell marker expression, prolonged the survival time of xenograft mice and reduced tumor size.^30^ Other tumors such as glioma,^7^ colon cancer^13^ and melanoma^31,32^ cells also exhibit this phenomenon when reprogrammed by defined transcription factors. On the basis of these studies, we conclude that SOX-2, OCT-4, KLF-4, Nanog and Lin-28A contribute greatly to the events of dedifferentiation.

Hypoxia as a physiological phenomenon exists in tumors.^33,34^ Although a few studies have paid attention to the influence of hypoxia on unsorted cancer cells,^15,16,35,36^ these studies could not identify the role of hypoxia on non-CSCs, because the increased expression of CD133, SOX-2 or OCT-4 under hypoxia may be from CSCs themselves. In addition, almost all the traditional studies used cancer cells cultured in stem cell medium containing EGF, FGF2 or IGF-1.^35^ This introduced another question of whether the increased expression of stem cells may be induced by the stimulation of EGF, FGF2 and IGF-1 and not hypoxia. To avoid these problems, we sorted CD133^−^ cells (A549, HepG2) or CD133^−^/CD15^−^/NESTIN^−^ cells (GL261) by MACS three times and cultured cells in DMEM/F12 medium without EGF, FGF2 or IGF-1. In our study, we think the most important and powerful result was the sphere formation by a single sorted cancer cell. The results showed that compared with the control cells under normoxic conditions, the sphere formation rate under hypoxia by a single cell was much higher than the proportion of CSCs in the glioma, lung cancer and hepatoma cells. Though some newly formed spheres in our study may come from CSCs, we think that a subset of spheres must come from some non-CSCs. To verify our results, we detected the expression of SOX-2, OCT-4, KLF-4, Nanog and Lin-28A under hypoxia and found that hypoxia promoted the expression of these factors remarkably. At the same time, we also demonstrated that CD133 expression increased significantly after hypoxia treatment in glioma, lung cancer and hepatoma cell lines. In addition, we found that these newly formed spheres exhibited asymmetric division, cell cycle arrest^15,37^ and a lower apoptosis rate,^38^ which was in accordance with the basic features of CSCs. Therefore, according to our *in vitro* results, we can conclude that cancer stem cells can be induced through dedifferentiation in glioma, lung cancer and hepatoma under hypoxia conditions. The existence of dedifferentiation shows us that there exists an interchange between non-CSCs and CSCs, which promote cancer to become more malignant; thus, we should take the dedifferentiation process into consideration in developing cancer treatments. However, the molecular mechanism of this dedifferentiation phenomenon under hypoxic conditions needs further study, and we speculate that SOX-2, OCT-4, KLF-4, Nanog and Lin-28A may have an important role in this process, based on traditional studies regarding the formation of iPS cells by these defined factors and the influences of these factors on tumors.

In summary, this study showed that cancer stem-like cells can be induced through dedifferentiation under hypoxic conditions in glioma, hepatoma and lung cancer, which provides a new theory of tumor development, recurrence and resistance to chemo- and radiotherapy. Hence, we should take non-CSCs and the hypoxic microenvironment into consideration when developing tumor treatments.

## Materials and Methods

### Cell culture and non-CSCs isolation

The GL261, A549 and HepG2 cell lines were bought from ATCC. The GL261 cells were cultured in DMEM/F12+10% fetal bovine serum (FBS), and the A549 and HepG2 cells were cultured in DMEM+10% FBS. CD133, CD15 and NESTIN were considered glioma stem cell markers and were used to sort CD133^−^CD15^−^NESTIN^−^ GL261 cells as non-GSCs. For A549 and HepG2 cells, we considered CD133 to be a stem cell marker and sorted CD133^−^ cells by magnetic cell sorting (MACS). The CD133^+^ immune magnetic bead separation kits were bought from Miltenyi Biotech, Bergisch-Gladbach, Germany. First, we collected cancer cells cultured under normoxia for 3 days in DMEM/F12+10% FBS at 37 °C, and we then used 0.25% trypsin to digest the cancer cells and obtain a cell suspension. Next, PBS containing 0.5% BSA and 0.08% EDTA (PBSE; 10^8^ cells/500 *μ*l) was used to re-suspend the cancer cells, and the cells were then incubated at 4 °C for 15 min with CD133^+^ IgGs (Miltenyi Biotech). Then, we centrifuged the cell suspension again and re-suspended cells in PBSE (10^8^ cells/300 *μ*l) and labeled the cells with IgG MicroBeads (Miltenyi Biotech) for 15 min at 10 °C. We next centrifuged the cell suspension and washed and re-suspended cells in PBSE. The miniMACS magnet was fixed on the MACS multistand, and 500 *μ*l PBSE was used to flush the cell separation column. Finally, the cell suspension was poured into the column reservoir, and we collected the unlabeled nonmagnetic cells as CD133^−^ cells. To increase the purity of the CD133^−^ cells, we repeated the above steps at least three times. Sorted A549 and HepG2 CD133^−^ cells were cultured in DMEM+10% FBS. For GL261 cells, we used a similar method to isolate the CD133^−^/CD15^−^/NESTIN^−^ cells and cultured them in DMEM/F12+10% FBS. All sorted cells were used within 1 week, and we sorted the non-CSCs again if these cells had been cultured for more than 7 days.

### Real-time quantitative PCR

Sorted GL261, A549 and HepG2 cells were cultured under hypoxia for 0, 3, 6, 9, 12 and 24 h; we then collected the cells and analyzed the expression of stem cell markers at the mRNA level. Total mRNA was extracted from the cells, and the expression of CD133, SOX-2, KLF-4, OCT-4, Nanog, Lin-28A, CD15 and NESTIN was examined by real-time quantitative PCR (RT-qPCR). The reactions were performed with an initial denaturation at 94 °C for 5 min, followed by 40 cycles of denaturation at 94 °C for 30 s, annealing at 57 °C for 30 s, and extension at 72 °C for 30 s. The primer sequences were as follows:[Table tbl1]

### Western blot analysis of stem cell markers

Proteins were collected from sorted cells after hypoxia exposure for 0, 12, 24, 48 and 72 h. We then subjected the proteins to SDS-PAGE and transferred them to nitrocellulose membranes, which were blocked with 5% non-fat milk and incubated with primary antibodies against CD133 (1 : 1000, MBS462020, MyBiosource, San Diego, CA, USA), SOX-2 (1 : 1000, MAB2018, R&D Systems, Minneapolis, MN, USA), KLF-4 (1 : 1000, Human: AF3640; Mouse: AF3158 R&D Systems), OCT-4 (1 : 1000, MAB1759, R&D Systems), Nanog (1 : 1000, Human: AF1997; Mouse: AF2729 R&D Systems), Lin-28A (1 : 1000, NBP1–49537, Novus Biologicals, Littleton, CO, USA), CD15 (1 : 1000, MAB2155, R&D Systems) or NESTIN (1 : 1000, MAB2736, R&D Systems). *β*-Actin was used as an internal control.

### Stem cell marker detection by immunofluorescence

Sorted cells were cultured under hypoxia for 48 h to detect the expression of CD133, SOX-2, OCT-4, Lin-28A, KLF-4, Nanog, CD15 and NESTIN. The sorted cell lines were exposed to hypoxia for 48 h, and 4% paraformaldehyde was used to fix the cells at 4 °C for 10 min. The cells were washed with PBS, and 10% serum in PBS containing 0.5% Triton X-100 was used to block the cells. The cells were then incubated for 24 h at 4 °C with primary antibodies against CD133 (1 : 150, MBS462020, MyBiosource, San Diego, CA, USA), SOX-2 (1 : 100, MAB2018, R&D Systems), KLF-4 (1 : 100, Human: AF3640; Mouse: AF3158; R&D Systems), OCT-4 (1 : 100, MAB1759 R&D Systems), Nanog (1 : 100, Human: AF1997; Mouse: AF2729 R&D Systems), Lin-28A (1 : 100, NBP1–49537, Novus Biologicals), CD15 (1 : 100, MAB2155, R&D Systems) or NESTIN (1 : 100, MAB2736, R&D Systems). The cells were then washed with PBS three times. Appropriate fluorophore-labeled secondary antibodies were added to the cells and incubated at 37 °C for 1 h. Images were acquired with a laser scanning confocal microscope (LSM780, ZEISS, Jena, Thuringia, Germany).

### Flow cytometric analysis

CD133, as a cancer stem cell marker, was detected in GL261, A549 and HepG2 cells under hypoxia by flow cytometry. Because of the query of CD133 as a glioma stem cell marker, we also detected the proportion of CD15- and NESTIN-positive GL261 cells after hypoxia exposure. Sorted cells were exposed to hypoxia for 0, 3, 6, 9, 12 and 15 days, then collected and incubated with an anti-CD133 antibody (Human: Miltenyi Biotech; Mouse: BioLegend, San Diego, CA, USA), anti-CD15 antibody (FAB2155G-100, R&D Systems) and anti-NESTIN antibody (IC2736P, R&D Systems) and analyzed with FCM. We also used flow cytometry to detect cell cycle arrest and apoptosis in the sorted cells under hypoxia exposure for 0, 1, 3, 5 and 7 days. Besides, spheres formed in hypoxia environment were also analyzed cell cycle; and cell apoptosis of the newly formed spheres was analyzed after temozolomide (100 *μ*M) treatment in normoxia.

### Clonogenicity and asymmetric division assays

Sorted cells were digested with 0.25% trypsin, centrifuged and then suspended in DMEM/F12+10% FBS. We then counted and diluted the cells to 1500 cells/1 ml DMEM/F12+10% FBS, and 1 *μ*l of the mixed medium was added to each well of 96-well plates containing 170 *μ*l serum-free DMEM/F12 medium. Six 96-well plates were grouped randomly into two groups; one group was incubated at 37 °C with 1% O_2_ and 5% CO_2_ and another was incubated at 37 °C with 21% O_2_ and 5% CO_2_ (Figure 4a). We observed, imaged and recorded the cell growth state at 0, 3, 7, 14 and 21 days. The newly formed spheres were centrifuged, and half of them were cultured with stem cell medium (DMEM/F12+EGF+FGF2+B27) and the others were incubated with differentiation culture medium (DMEM/F12+10% FBS). Both groups of spheres were cultured at 37 °C with 21% O_2_ and 5% CO_2_ and the cell state was recorded at day 1, 3 and 5.

### Statistical analysis

SPSS 19.0 was used for the statistical analysis, and the data were presented as the mean±standard deviation. The differences between cells cultured in hypoxia and normoxia were evaluated with a one-sample *t*-test or paired-samples *t*-test. A *P*-value less than 0.05 was considered statistically significant.

## Figures and Tables

**Table 1 tbl1:** 

	Upstream (5′→3′)	Downstream (5′→3′)
Human SOX-2	GGAGGGGTGCAAAAGAGGAGAG	TCCCCCAAAAAGAAGTCCAGG
Human OCT-4	CCCGCCGTATGAGTTCTGTGG	CCGGGTTTTGCTCCAGCTTCTC
Human KLF-4	GGCTGCGGCAAAACCTACAC	CGGGCGAATTTCCATCCAC
Human Nanog	CCGCGCCCTGCCTAGAAAAGAC	AGCCTCCCAATCCCAAACAATACG
Human CD133	GCCCCCAGGAAATTTGAGGAAC	GCTTTGGTATAGAGTGCTCAGTGATTG
Human Lin-28A	GGCGGGAGGGTAGGAAAGC	CAGCAAAATCAACCATCAAATAAAC
Mouse SOX-2	GGGGGCAGCGGCGTAAGATG	CCCGCTCGCCATGCTGTTC
Mouse OCT-4	GCCCGGAAGAGAAAGCGAAC	GGGGCAGAGGAAAGGATACAG
Mouse KLF-4	CCGGCCCAACACACACGACTTC	GAACCCGGTGGCATGAGCTCTTG
Mouse Nanog	GCCCAGCTGTGTGCACTCAAGG	GGCTTCCAGATGCGTTCACCAGATA
Mouse Lin-28A	GGGGTGGGGAGTTTCGTTTACATG	GGGGAGAGGGAGACAAGAAACCAAG
Mouse CD133	CCGCGATGGACTCTGCTGTTAATG	GGGCACAGTCTCAACATCGTCGTATAC
Mouse CD15	ATCGGGCTGCTGCACACTG	AGCGGAAGTAGCGGCGATAGAC
Mouse NESTIN	GCCCAAGCAGGTGAACAAGACT	CAGCCCTTGCATTCCAGAGTCT
*β*-Actin	ACCCGCCGCCAGCTCACC	GGGGGGCACGAAGGCTCATC
