# Supplementary material for: Cancer stem-like cells can be induced through dedifferentiation under hypoxic conditions in glioma, hepatoma and lung cancer
Source: Cell Death Discov. 2017 Jan 23;3:16105–. doi: 10.1038/cddiscovery.2016.105 (PMC5253691; doi:10.1038/cddiscovery.2016.105)
Supplement: Supplementary Figures [file cddiscovery2016105-s1.doc]

**Supplementary figure description**

**Sup Figure S1** The RT-qPCR analysis showed an up-regulation in the expression of SOX-2, OCT-4, KLF-4, Nanog, Lin-28A and CD133 in a time-dependent manner in sorted GL261 and HepG2 cells under hypoxic conditions (^*^*P*<0.05).

**Sup Figure S2 A-B** Western blot analysis showed that the transcription factors and stem cell markers were not expressed under normoxia in GL261 and A549 cells. However, there was an increase in the expression of SOX-2, OCT-4, KLF-4, Nanog, Lin-28A and CD133 in a time-dependent manner after hypoxia treatment (^*^*P*<0.05). **C** Flow cytometry detected the cell cycle of newly formed spheres in hypoxia from sorted GL261, A549 and HepG2 cells arrested in G_0_/G_1_ phase (^*^*P*<0.05); **D** The cell apoptosis rate was much lower in spheres compared with control after temozolomide treated in normoxia (^*^*P*<0.05).
