# Supplementary figures and images for: Cancer stem-like cells can be induced through dedifferentiation under hypoxic conditions in glioma, hepatoma and lung cancer
Source: Cell Death Discov. 2017 Jan 23;3:16105–. doi: 10.1038/cddiscovery.2016.105 (PMC5253691; doi:10.1038/cddiscovery.2016.105)

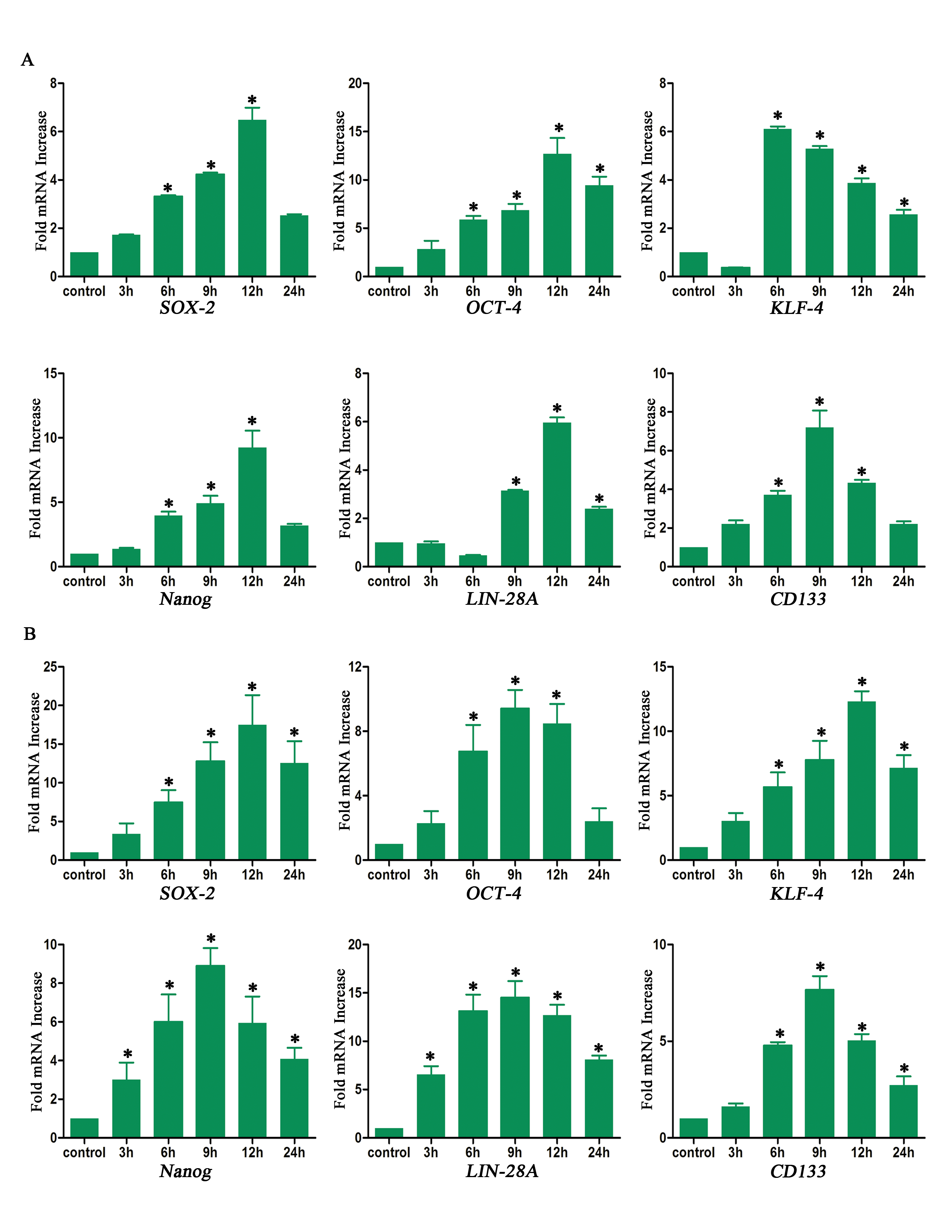

Supplement: Supplementary Figure S1 [file cddiscovery2016105-s2.tiff]

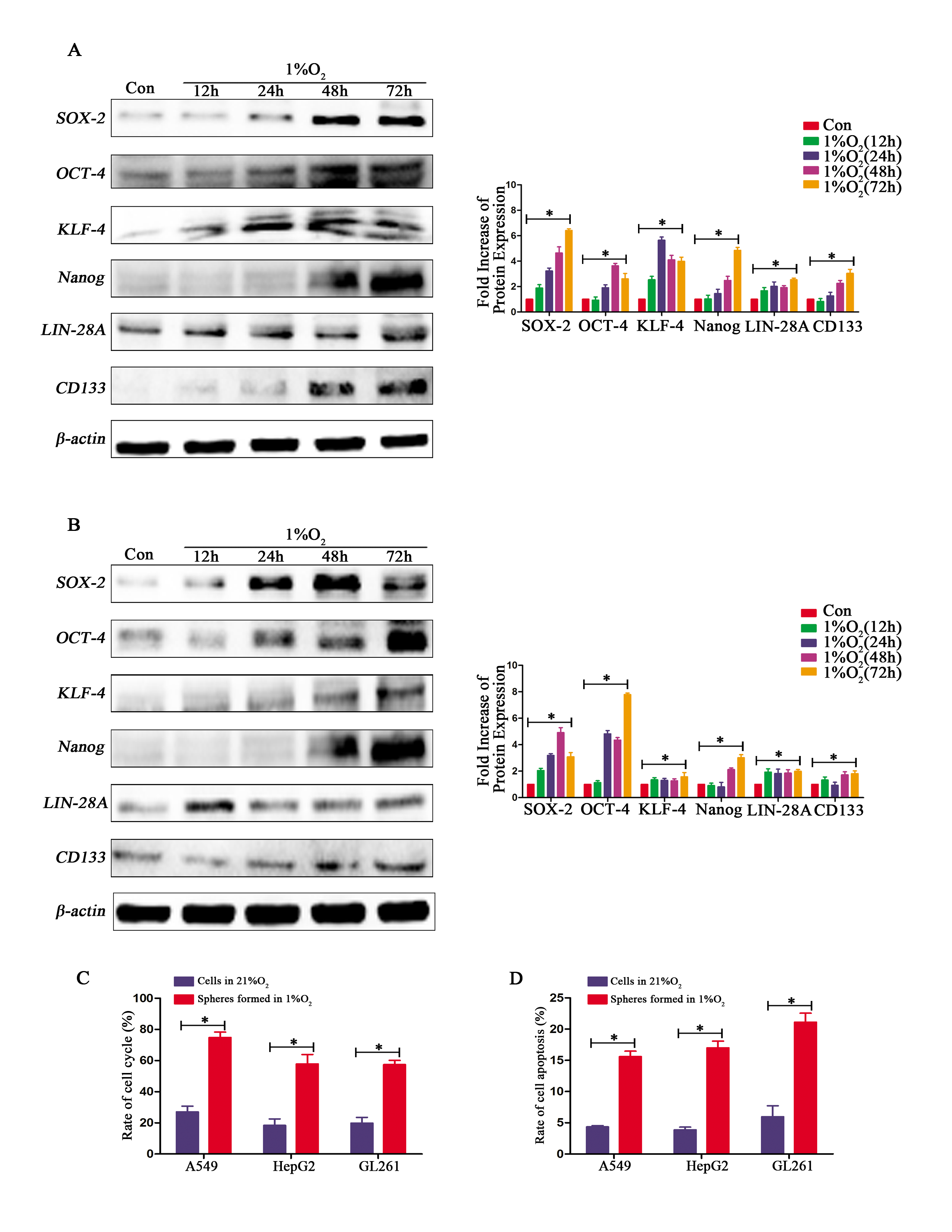

Supplement: Supplementary Figure S2 [file cddiscovery2016105-s3.tiff]
